# Evaluating Sensory Integration/Sensory Processing Treatment: Issues and Analysis

**DOI:** 10.3389/fnint.2020.556660

**Published:** 2020-11-26

**Authors:** Stephen Camarata, Lucy Jane Miller, Mark T. Wallace

**Affiliations:** ^1^Department of Speech and Hearing Sciences, Bill Wilkerson Center, Vanderbilt University School of Medicine, Nashville, TN, United States; ^2^STAR Institute for Sensory Processing, Greenwood Village, Centennial, CO, United States; ^3^School of Medicine, University of Colorado, Denver, CO, United States; ^4^Graduate School, Vanderbilt University, Nashville, TN, United States

**Keywords:** sensory integration, sensory processing disorder (SPD) intervention, behavioral intervention, treatment effect analysis, naturalistic behavioral intervention

## Abstract

For more than 50 years, “Sensory Integration” has been a theoretical framework for diagnosing and treating disabilities in children under the umbrella of “sensory integration dysfunction” (SID). More recently, the approach has been reframed as “the dimensions of sensory processing” or SPD in place of SID, so the review herein describes this collective framework as sensory integration/sensory processing treatment (SI/SP-T) for ASD. This review is not focused on diagnosis of SI/SPD. Broadly, the SI/SPD intervention approach views a plethora of disabilities such as ADHD, ASD, and disruptive behavior as being exacerbated by difficulties in modulating and integrating sensory input with a primary focus on contributions from tactile, proprioceptive, and vestibular systems which are hypothesized to contribute to core symptoms of the conditions (e.g., ASD). SI/SP intervention procedures include sensory protocols designed to enhance tactile, proprioceptive, and vestibular experiences. SI/SP-T procedures utilize equipment (e.g., lycra swings, balance beams, climbing walls, and trampolines), specific devices (e.g., weighted vests, sensory brushes) and activities (e.g., placing hands in messy substances such as shaving cream, sequenced movements) hypothesized to enhance sensory integration and sensory processing. The approach is reviewed herein to provide a framework for testing SI/SP-T using widely accepted clinical trials and event coding methods used in applied behavior analysis (ABA) and other behavioral interventions. Also, a related but distinct neuroscientific paradigm, *multisensory integration*, is presented as an independent test of whether SI/SP-T *differentially* impacts sensory integration and/or multisensory integration. Finally, because SI/SP-T activities include many incidental behavioral events that are known as developmental facilitators (e.g., contingent verbal models/recasts during verbal interactions), there is a compelling need to control for confounds to study the *unique* impact of sensory-based interventions. Note that SI/SP-T includes very specific and identifiable procedures and materials, so it is reasonable to expect high treatment fidelity when testing the approach. A patient case is presented that illustrates this confound with a known facilitator (recast intervention) and a method for controlling potential confounds in order to conduct unbiased studies of the effects of SI/SP-T approaches that accurately represent SI/SP-T theories of change.

## Overview: Sensory Integration/Sensory Processing Treatment (SI/SP-T) for ASD Is A Widely-Implemented Intervention Approach but with An Emerging but Limited Evidence Base

The goal of this article is to provide a review of sensory integration/sensory processing treatment (SI/SP-T) in Autism Spectrum Disorder (ASD), an intervention used widely in schools and clinics, to generate a framework and pedagogy for systematically testing behavioral interventions for children with disabilities. That is, we view SI/SP-T as one of several potential interventions for children with developmental disabilities which can be evaluated using widely accepted evidence-based standards and which can be objectively tested using clinical trial approaches to optimize an intervention for children with disabilities. Because there is considerable variation in nomenclature, and many researchers and clinicians have shifted from using “sensory integration” to “sensory processing,” (see Miller et al., 2009) we will be including both of these terms designated as “SI/SP-T” in our review. This combination is utilized because the term “sensory integration” continues to be included in the literature and in clinical practice along with the term “sensory processing.” Large scale intervention studies are needed because, despite widespread implementation, particularly for children with Autism Spectrum Disorder (ASD), Down Syndrome, attention deficit hyperactivity disorder (ADHD), and other developmental disabilities, SI/SP-T has an emerging but limited evidence base in the literature (see, for example, Pfeiffer et al., 2018), necessitating additional large-scale studies. Therefore, the review herein will include a description of the origins of SI/SP-T, current evidence, considerations for conducting fair clinical trials, a review of how to control for potential cofounds, a description of how to test for generalized changes in SI/SP using *multisensory* integration approaches, a case example of how confounds can impact clinical intervention studies of SI/SP-T, suggestions for future research directions, and clinical implications.

## Evidence-Based Practice: Levels of Evidence

There have long been universal protocols for evaluating treatment efficacy and effectiveness in medicine and in behavioral interventions (Reynolds, 2008). These procedures arose, in part, from the long-standing persistence of treatments in clinical settings that, when tested fairly, proved to be ineffective or even harmful. For example, chelation, an established biomedical treatment for acute exposure to lead and other toxic metals, was hypothesized to be an effective “detox” for children with ASD (see James et al., 2015). This treatment was based on an unproven presumption that because ASD was caused, at least in part, by exposure to mercury, chelation would improve autism symptoms (see Davis et al., 2013). Moreover, there have been many testimonials and qualitative case studies suggesting that the approach was effective. But, when tested using clinical trials, chelation not only failed to improve symptoms of ASD, but also caused adverse reactions, including death, in some cases (Baxter and Krenzelok, 2008). Of course, the overwhelming majority of treatments for autism do not include death as a potential side effect, but there are certainly many treatments that despite having limited data that conform to evidence-based practice guidelines (Weiss et al., 2008; Guldberg, 2017), are nonetheless widely implemented.

It must be stated explicitly that a limited evidence base **does not mean that a treatment is ineffective;** when tested, an emerging treatment may subsequently be validated when large scale studies are conducted. However, ethical practice guidelines include preferentially delivering treatments that currently have credible evidence over those that do not. There is an extensive evidence base showing moderate to large effect sizes for improving a wide range of ASD symptoms using behavioral intervention procedures that do not directly target SI/SP (e.g., Naturalistic Developmental Behavioral Interventions, NDBI; see Sandbank et al., 2020). That is, SI/SP-T can be conceptualized and tested as a naturalistic behavioral intervention and conditions such as ASD can yield fair tests of the approach. Because of this, within the framework of widely used treatment efficacy and effectiveness evaluation procedures that include group and single case (single subject) designs, emerging approaches require systematic evaluation and levels of evidence that meet or exceed those of existing interventions (e.g., NDBI) to be included in validated treatment options.

Broadly, evidence-based rubrics classify “evidence” along a weak to strong continuum (see Brighton et al., 2003). The lowest level of evidence includes *case presentations* and *case series* studies. These are descriptive and often include qualitative indices such as goal attainment scaling with limited or no experimental control of bias. It should be noted, however, that these studies are indeed evidence and that there have been important discoveries that originated with case reports and case series studies. On the other hand, a lack of control and potential for bias impacting results, are considered weak evidence (Brighton et al., 2003) and there have been many treatments that showed initial promise in case reports that did not prove beneficial when more controlled studies were completed. *Case-control* studies are similar to case reports and case series studies but include a control/comparison patient (or patients). Although most are retrospective (a group of similar patients wherein some improved and some did not), this approach can yield even stronger evidence when implemented as prospective single subject/single case design control procedures (see Kennedy, 2005; Maggin et al., 2019). The next highest level of evidence includes prospective *cohort studies*, which essentially can be used to determine whether there are differential pre-post- gains in qualitative and/or quantitative benchmarks such as goal attainment scaling and standardized assessments. These also include limited or no experimental control of bias but are quite useful. The next level, *randomized control trial* (*RCT*), is considered the highest level of evidence when randomization and blinding are implemented. Unblinded and/or subjective qualitative RCTs (e.g., Goal Attainment Scaling) are viewed as credible evidence, but weaker than blinded RCTs. The “ultimate” level of evidence includes a meta-analysis of aggregated strong RCTs showing consistently meaningful effect sizes across studies. Our analysis of SI/SP-T in ASD is predicated on this widely used evidence rubric. Bear in mind that patient and clinician testimonials are not considered evidence.

## Origins of SI/SP-T: A Brief Overview of Sensory Integration/Sensory Processing Treatment Approaches

Ayres (1972, p. 4) described sensory integration dysfunction as a problem in the ability to “organize sensory information for use” and along with motor performance, as a key element of intervention (see also Ayres, 1963; Ayres and Robbins, 2005). In addition to her clinical work, Ayres published many studies focused on the assessment and treatment of SI, and she developed assessments for SI (e.g., Ayres, 1989, 1996). Ayres’ definition encompasses a broad range of behaviors and includes disruptions in social interaction and behavioral regulation (Miller et al., 2007a). While acknowledging that many sensory-based approaches incorporate motor performance in accord with Ayres’ framework (Ayres, 1979), we will be focusing the review on sensory parameters. A recent definition of SI derived from a nosology of sensory integration disorder includes “difficulty detecting, modulating, interpreting and/or responding to sensory experiences, which is severe enough to disrupt participation in daily life activities and routines and learning” (Miller et al., 2007a). Several subtypes are proposed in one or more sensory systems, including auditory, visual, gustatory (taste), olfactory (smell), somatosensory (proprioception and touch), vestibular, and interoceptive (the sense involved in the detection of internal regulation, such as heart rate, respiration, hunger, and digestion) domains. In 2009, Miller et al. (2009) suggested a change in nomenclature from “sensory integration” to “sensory processing” disorder while maintaining the foundational sensory elements. Thus, these eight sensations are the central targets of many SI/SP-T sessions. Moreover, SI/SP-T is posited to directly improve attentional, emotional, motoric, communication, and/or social difficulties (see Miller et al., 2014). Difficulty in sensory integration/sensory processing is hypothesized to result in challenges related to initiating or sustaining peer interactions, developing engaged relationships, participating in activities of daily living, and regulating arousal behaviors. Specific developmental domains, such as language development (e.g., Ayres and Mailloux, 1981; Mauer, 1999), are also hypothesized to be impacted and to thus incidentally benefit from SI/SP-T. The impact of these sensory parameters on quantitative indices of domains such as language development is directly testable using well-established experimental approaches.

Within this theoretical framework, common manifestations of sensory integration/sensory processing deficits in children with developmental disabilities, such as ASD and ADHD when sensory symptoms are displayed including responses to stimulation more quickly, more intensely, and for a longer duration than do typically developing individuals. It should be noted that SI/SPD is not exclusive to ASD, ADHD or any other developmental condition and not every child with ASD, ADHD or any other developmental condition should be diagnosed with SI/SPD. Examples in everyday life include extreme responses to stimuli such as noise in a classroom, odors in a restaurant, the touch of clothing, the clipping of finger and toenails, the movement of playground equipment, and/or the sight of cluttered environments. Behavioral responses are proposed to include a range of “fight, flight or freeze” reactions such as aggression, withdrawal, or preoccupation with the expectation of sensory input. Secondary social effects seen in preschoolers with SI/SPD include severe difficulty forming and maintaining peer relationships and/or extreme efforts to control events in the environment by over-reliance on routines. Hypothesized correlates include profound behavior regulation problems, including temper tantrums, outbursts, hitting, kicking, biting, spitting, and other maladaptive behaviors, and profound withdrawal from groups.

Additionally, preschool children with SI/SPD are also reported as being slow to respond to sensation, showing reduced or absent responses, and/or requiring more intense stimuli to respond to the demands of the situation. Examples include not responding to one’s name being called and failing to notice when hurt, thirsty, or hungry (see the examples in Miller et al., 2014). Some children with SI/SPD are also reported to have an insatiable need for sensation, well beyond that which is typical, often to the extent that safety is a concern. These children derive great pleasure from “crashing and falling” and have great difficulty sitting still. Parents and peers may describe such children as being “in my face and in my space,” “constantly touching people or objects,” and demanding significant time and attention (Miller et al., 2007a; Ben-Sasson et al., 2019). These impulsive and hyperactive behaviors may adversely impact student outcomes. Lastly, preschool children with SI/SPD present with motor delays sometimes categorized as “associated symptoms” (Ming et al., 2007) that are purportedly due to an underlying impairment in the ability to interpret sensations (Roley et al., 2015). Examples include difficulty initiating, planning, sequencing, and building repertoires of action plans, all of which are essential to motor planning to accomplish multi-step daily routines. This SI/SPD framework is often applied to symptoms of conditions such as ASD when delivering SI/SP-T. But it is important to note that the aforementioned features of ASD have also been addressed *without* utilizing sensory activities so that there are alternative perspectives as to the nature and extent of SI/SP features in ASD interventions (see the review and meta-analysis in Sandbank et al., 2020).

Thus, despite widespread implementation of SI/SP-T based services, there is an extensive portion of the assessment and intervention literature for children with disabilities that does not interpret these behaviors through the lens of sensory integration or sensory processing, relying instead upon another operant/applied behavioral analysis and/or physiological foundations (as examples, see Sappok, 2019; Sandbank et al., 2020). Theoretically motivated, hypothesis-driven studies within the context of fair clinical trials of SI/SP-T are needed to resolve this disparity in the theoretical ontogeny of sequelae of developmental disabilities such as ASD. This will shed light on best practices for intervention in conditions such as ASD. Moreover, there continues to be considerable heterogeneity in the field regarding treatment and the underlying theories driving these interventions (see for example, Sandbank et al., 2020). Importantly, the “fair evaluation” of an intervention must be faithful to the implied or explicit theory of change for that intervention. Because of this, it is important to briefly review a representative theory of change for SI/SP-T.

## Theory of Change for Sensory Integration/Sensory Processing Treatment

Hundreds of publications have described SI/SP-T since 1964, though the literature continues to contain relatively few large-scale randomized trials directly testing the intervention (Ayres, 1972; Kimball, 1993; Kinnealey and Miller, 1993; Parham, 1998; Miller et al., 2001, 2007b; Bundy et al., 2002; Pfeiffer et al., 2011, 2018; Schaaf et al., 2014, 2018). Most of the literature on this topic includes inconsistent terminology between studies as well as limited high-quality evidence, and design limitations (see Miller et al., 2007c; Schaaf et al., 2018). Additionally, because authors often utilize terminology, theoretical constructs, and observational frameworks that are inconsistent (see Schaaf and Davies, 2010), it can be difficult to aggregate studies and to specify consistent outcome measures. Thus, although some studies provide credible evidence of treatment effects, SI/SP-T does not yet have a strong evidence-base. For example, Schoen et al. (2019) conducted a systematic review of Ayres Sensory Integration (ASI) treatment and found only two studies that met a majority of quality indicators and one additional study that met a “plurality” of quality metrics. In contrast, reviews of NBDIs include dozens or even hundreds of studies (e.g., Sandbank et al., 2020). For purposes of this review, we are using the SI/SP-T nosology by Miller et al. (2007a), and we have adapted the conceptual theory of change from Miller et al. (2001) as an example of a testable SI/SP-T framework (see Table 1). To be sure Ayres Sensory Integration (e.g., the review of ASI in Watling and Hauer, 2015; Schoen et al., 2019) or any other well-defined approach within the broad rubric of SI/SP-T could also be tested, we utilize the framework of Miller et al. (2001) herein as an example of how this can be accomplished.

**Table 1:** Hypothesized social and behavioral effects of sensory disruptions.

Dimensions	Behaviors observed
**Sensory symptoms** Results in 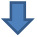	Difficulty regulating sensory input: over or under responsivity (Tactile, Movement, Taste, Smell, Auditory, or Visual stimuli); difficulty interpreting internal sensations (body awareness, interoception), and difficulty discriminating external sensations (from the environment).
**Motor symptoms** Results in 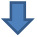	Poor coordination, Clumsiness, Awkwardness, Poor posture, Limited planning and sequencing of motor skills; Inability to perform multistep tasks.
**Behavioral symptoms** Results in 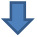	Aggression, Anger, Dysregulation, Tearfulness, Withdrawal. Anxiety, Poor attention, Hyperactivity, Poor impulse control.
**Social symptoms**	Social isolation, Withdrawal, Poor social relationships with peers and adults, Discomfort in social situations.

**Figure 1:**
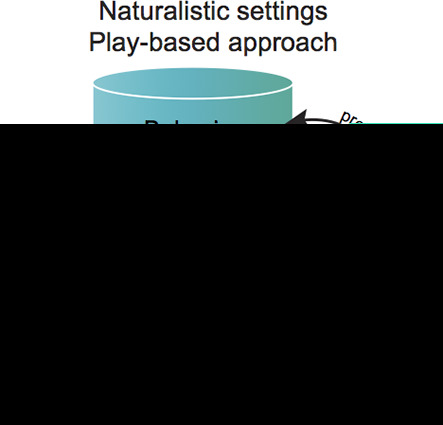
Theory of change for sensory integration/sensory processing (SI/SP) therapy.

The model in Figure 1 suggests that sensory function is foundational to motor ability, social skill, and a broad range of behavior. Thus, when a disruption occurs in sensory abilities (including disruption in modulation, discrimination, and integration of sensory input), testable cascading effects are posited for several “higher-level” domains, such as social skills. These disruptions are believed to translate to problems with participation at home, at school, and in the community (see Table 1). A Model of Change using SI/SP-T as articulated above relates to proposed changes in motor, social, and behavioral challenges. It is noteworthy that SI/SP-T can be implemented in a manner that is consistent with the model within the context of a blinded RCT with primary and tertiary measures of hypothesized effects. Thus, *the SI/SP-T theory of change* can be measured using a *fidelity of treatment scale* following evidenced-based standards for all behavioral interventions. The structure and delivery of SI/SP-T are founded on the incorporation of tactile (touch), proprioceptive (pressure, position, and muscle exertion), and vestibular (movement and balance) activities in a naturalistic, play-based intervention session. These sensory events can all be operationally defined and reliably measured using observational coding.

For an intervention to be evaluated fairly, these enhanced sensory integration experiences must be selected specifically to fulfill the needs and behaviors of the individual child and measured systematically. For example, if a child displays an unusual sensory profile marked by tactile over-responsivity, then SI/SP-T activities should provide systematic exposure to different tactile sensations (Miller et al., 2014). Systematic exposure to tactile activities is hypothesized to not only decrease tactile over-responsivity but also to improve the behaviors and skills disrupted by tactile over-responsivity, which can all be measured objectively using event coding and/or rating scales. Again, each of these links changes be tested directly.

Additionally, SI/SP-T is hypothesized to benefit children with reduced tactile discrimination. A child who does not interpret (discriminate) tactile sensations delivered to her fingers, hands, and feet, may have trouble participating in activities requiring accurate tactile interpretation (e.g., difficulty buttoning, writing, and manipulating small objects). Again, this functional relationship is testable.

## Testing Behavioral Treatments

For this review, behavioral treatment is defined broadly as interventions that employ clinician-child or parent-child interaction *excluding* pharmacological agents (e.g., as in Hampton and Kaiser, 2016). This includes naturalistic play-based interventions and highly structured operant conditioning treatment methods (Sandbank et al., 2020). Although some have argued that only operant “discrete trials” should be identified as “behavioral” or exclusively falling within the scope of “applied behavioral analysis,” behavioral interventions have long been extended to include play-based “naturalistic” treatments (McLean and Snyder-McLean, 1978). As an example, Sid Bijou, one of the founders of the applied behavioral analysis field, adapted Kantor (1977) linguistic theory for study within a behavioral rubric, including conversational elements (see Bijou et al., 1986; Ghezzi, 2010). This framework has been widely applied to study conversational based interventions (see as examples, Koegel et al., 1987; Camarata, 1993; Camarata et al., 1994; Gillum and Camarata, 2004). Table 2 provides a theory of change for a naturalistic behavioral intervention (Pivotal Response Training, Koegel et al., 2016) within a behavioral framework. The key point herein is that SI/SP-T can be examined—and tested—within a behavioral framework similar to those applied for naturalistic interventions (e.g., NDBIs).

**Table 2:** Elements of an example transactional “ABA” treatment (pivotal response teaching).

**CUE**
**Child attention**
Gain child’s attention before providing cue
**Clear and appropriate**
Provide related, clear and developmentally appropriate cues
**Child choice**
Allow child a choice of activity or materials
**Take turns**
Take turns by modeling appropriate behavior
**Maintenance tasks**
Intersperse tasks the child has already mastered
**Multiple cues**
Provide cues that require responding to multiple elements
**Child behavior (correct, incorrect, and attempt)**
**RESPONSE**
**Contingent**
Provide appropriate consequences based on child’s behavior
**Direct reinforcement**
Provide reinforcement directly related to the child’s behavior
**Good trying**
Reinforce child’s goal directed attempts

## Current Evidence Base for SI/SP Treatment

Given the widespread delivery of SI/SP based assessment and treatment, one would expect an extensive strong evidence base in the literature. Before delving into the current evidence on SI/SP-T, it is important to mention that practices are often widely provided to students with disabilities even in the absence of extensive supporting data-driven evidence. As an example, music therapy is a very common approach provided to children with ASD despite its currently limited evidence base (see Lense and Camarata, 2020). **Although problematic, an absence of evidence, unto itself, cannot be construed as invalidating**.

Our review indicated that to date, there have been small scale studies of several isolated sensory-based *procedures*, such as weighted vests or “brushing” programs, which usually suggest the procedures are not effective (e.g., Lang et al., 2012; Taylor et al., 2012). And there are a limited number of studies showing positive effects on goal attainment scaling (see the reviews in Schaaf et al., 2018; Schoen et al., 2019). But there are also several systematic reviews indicating inconsistent, weak, and/or inconclusive evidence. For example, Lang et al. (2012) reported, “Overall, three of the reviewed studies suggested that SI/SP-T was effective, eight studies found mixed results, and 14 studies reported no benefits related to SI/SP-T” (p. 1004). The majority of the studies reviewed by Lang et al. (2012), however, tested only one sensory-based procedure (e.g., a weighted vest or sensory brushing) but not a comprehensive form of SI/SP-T, in which a multi-component *approach* is implemented. **Thus, a fair test of SI/SP-T necessitates the delivery of multiple elements rather than piecemeal testing of isolated sensory-based procedures and tools** (e.g., wearing a weighted vest).

A critical review published in *Pediatrics* provides a comprehensive view that more accurately represents the treatment (Johnson and Myers, 2007): “The goal of [SI/SP-T] is not to teach specific skills or behaviors but to remediate deficits in neurologic processing and integration of sensory information to allow the child to interact with the environment more adaptively.” This perspective is highlighted in a recent review by Case-Smith et al. (2015) who concluded:

Studies of sensory-based interventions suggest that they may not be effective. However, these studies did not follow recommended protocols or target specific sensory processing problems. Although small randomized controlled trials resulted in positive effects for [SI/SP-T], additional rigorous trials using *manualized protocols* for [SI/SP-T] are needed to evaluate effects for children with [ASD] and sensory processing problems (p. 133).

As these reviews demonstrate, there is currently, at best, an emerging, but limited evidence base on SI/SP-T, with few positive outcomes and some null or negative outcomes.

Moreover, the current state of the evidence for SI/SP-T is accurately characterized in a review by the American Academy of Pediatrics (2012): “… the amount of research regarding the effectiveness of [SI/SP-T] is limited and inconclusive” (p. 1186). More recently, Weitlauf et al. (2017) reported in a follow-up review:

Some interventions may yield modest short-term (<6 months) improvements in sensory and ASD symptom severity-related outcomes; the evidence base is small, and the durability of the effects is unclear. Although some therapies may hold promise, substantial needs exist for continuing improvements in methodologic rigor (p. 347).

Moreover, recent meta-analyses and systematic reviews have consistently highlighted: (a) the paucity of intervention studies in SI/SP-T; and (b) a crucial need for credible intervention studies of SI/SP-T (see Sandbank et al., 2020). As an example, Pfeiffer et al. (2018) conducted a systematic review of SI/SP-T that yielded five articles meeting inclusion criteria and concluded “Because the number of studies that measured sensory processing or SI challenges were limited, researchers are encouraged to include these measures in future research to understand the impact of a broader range of cognitive and occupation-based interventions” (Pfeiffer et al., 2018, p. 1). Similarly, Pingale et al. (2020) reported “occupational therapists (OTs) use sensory diets to manage sensory processing disorder in children. The current evidence is limited. Also, the findings of the studies on the effects of sensory diets are mixed” (Pingale et al., 2020, p. 1). Schaaf et al. (2018) reviewed five studies and reported that “The evidence is strong that ASI [Ayres Sensory Integration] demonstrates positive outcomes for improving individually generated goals of functioning and participation as measured using Goal Attainment Scaling for children with autism,” but also reported that “Child outcomes in play, sensory-motor, and language skills and reduced caregiver assistance with social skills had emerging but insufficient evidence” (Schaaf et al., 2018, p. 1). In sum, large scale clinical trials are needed because there is evidence that SI/SP-T can improve “near point” proximal measures using qualitative Goal Attainment Scaling, but definitive outcomes for broader objective measures are less clear.

Despite a consensus in the literature on the need for additional evidence, SI/SP-T is currently widely implemented in schools by occupational therapists, speech-language pathologists, and other related services personnel (see McIntyre and Zemantic, 2017). For example, Devlin et al. (2011) recently reported that SI/SP-T using Ayres Sensory Integration Approach was one of the most prevalent intervention models in schools, which substantiates previous research findings (Spitzer et al., 1996; Case-Smith and Miller, 1999; Watling et al., 1999; Roley et al., 2001). A survey of occupational therapists revealed that 82% of respondents reported that they “always” use sensory-based treatment when working with children with ASD (Watling et al., 1999). Fifty-six percent of parents of children who received applied behavior analysis (ABA) treatment noted that their children with ASD had been exposed to sensory treatment as well (Smith and Antolovich, 2000, p. 1304; see also McIntyre and Zemantic, 2017). There is no doubt that sensory integration procedures have gained widespread popularity despite the ongoing need for a stronger evidence base. Given that SI/SP-T is “testable” within an evidence-based framework, further research is warranted to determine the efficacy of the approach (see Baker et al., 2008). The following sections describe approaches that could potentially strengthen the evidence base for SI/SP-T if the results of clinical-translational studies reveal unique effects for SI/SP-T.

## (Multi)Sensory Perception as A Window into SI/Sp-T: Multisensory Integration as A Distal Measure of The Impact of Sensory-Based Treatment

Multisensory integration is defined as the study of how the brain integrates and interprets input from multiple unisensory systems (Alais et al., 2010). The overlap in nomenclature with sensory integration/sensory processing may be confusing to clinicians and researchers. Multisensory integration differs from sensory integration/sensory processing in that it does not include intervention recommendations or downstream sequelae of disability while specifically focusing on tightly designed neural and cognitive studies of how specific primary sensory streams are integrated in real-time (e.g., auditory and visual). Studies of multisensory integration often elicit unisensory responses from two or more primary senses (e.g., audition and vision) and then compare the separate responses to effects observed when the inputs are combined (see Stevenson et al., 2014). If the core tenant of SI/SP-T is accurate, namely that SI/SP-T enhances sensory integration, multisensory integration provides a strong test of generalized effects of treatment explicitly designed to *improve* sensory integration. The literature on ASD provides an example of how one can expect distal multisensory impacts if SI/SP-T is delivered and the theory of change is accurate. As noted above, Sensory Integration Theory and practice was originated by Ayres (1972). *Multisensory Integration*, a branch of contemporary neuroscience devoted to understanding how the brain synthesizes information from the different sensory systems, establish striking behavioral and perceptual benefits derived from multisensory inputs (see Stein, 2012) and may provide a neurological test of SI/SP-T.

Although the terms “sensory integration” and “multisensory integration” have divergent theoretical and empirical origins, the hypothesized theory of change for the SI/SP-T approach is directly predicated on disruptions in the ability to integrate sensory and *multisensory* information. Consequently, *multisensory integration assessment* is hypothesized to be a useful distal, quantitative approach for testing this aspect of the SI/SP-T approach. Recent studies are developing highly effective methods for characterizing multisensory integration in developing children (Neil et al., 2006; Stephen et al., 2007; Hillock et al., 2011; Hillock-Dunn and Wallace, 2012), and some studies are focused on children with ASD. While there is a strong conceptual link between sensory integration and multisensory integration, there has not as yet been a systematic study of whether sensory-based treatment procedures have an incidental effect on multisensory integration. Indeed, sensory-based treatments are specifically designed to increase inputs from multiple sensory sources, which would facilitate learning and improve behavior *as a result of improved multisensory integration as a consequence of the sensory-based treatment*. Although therapists and teachers across many disciplines often incidentally incorporate information from multiple sensory modalities during treatment in the absence of targeted sensory integration procedures, sensory-based treatments specifically focus on delivering elements across different sensory systems. This approach of providing input from multiple sensory modalities is believed to benefit students by facilitating *multisensory integration*.

Ayres (1972) proposed that multisensory systems play a critical role in establishing a foundation upon which “higher-level” development can occur. Indeed, sensory and multisensory representations are viewed as forming the “building blocks” upon which higher cognitive abilities and learning can occur. However, any social/behavioral intervention, including sensory-based treatment, must ultimately be founded upon a series of empirically tested and validated procedures (Devlin et al., 2011). The strength of these multisensory integration assessments as distal outcome measures lies in the fact that SI/SP-T, if valid, should have a *differential* significant impact on MSI as compared to *nonsensory* comparison intervention conditions which do NOT include direct sensory-based treatment. Thus, a comparison of multisensory abilities between SI/SP-T and fair nonsensory behavioral treatment groups may be used to assess the specificity of treatments aimed at improving multisensory function. As an example, the aforementioned NDBI recast communication therapy approach yields strong effects on language, but, hypothetically should NOT improve MSI whereas SI/SP-T is hypothesized to improve language *and* MSI.

Tests that specifically index multisensory function are becoming increasingly important tools to provide an empirical evaluation of the integrity of sensory processing in individuals with disabilities (see Kwakye et al., 2011). Much of the work to date has focused on testing the ability to detect and discriminate sensory stimuli—both within and across different sensory modalities—in children and adults with disabilities compared to those considered “typically developing.” This work has revealed substantial differences in the manner in which individuals with disabilities, specifically ASD and dyslexia, integrate auditory and visual information. Therefore, there is a strong rationale for including multisensory assessments in future evaluations of the differential impact of SI/SP-T on individuals with ASD or who are typically developing as a direct link in the theory of change for sensory-based treatment approaches.

### Example From ASD and Multisensory Auditory-Visual Integration

**Figure 2:**
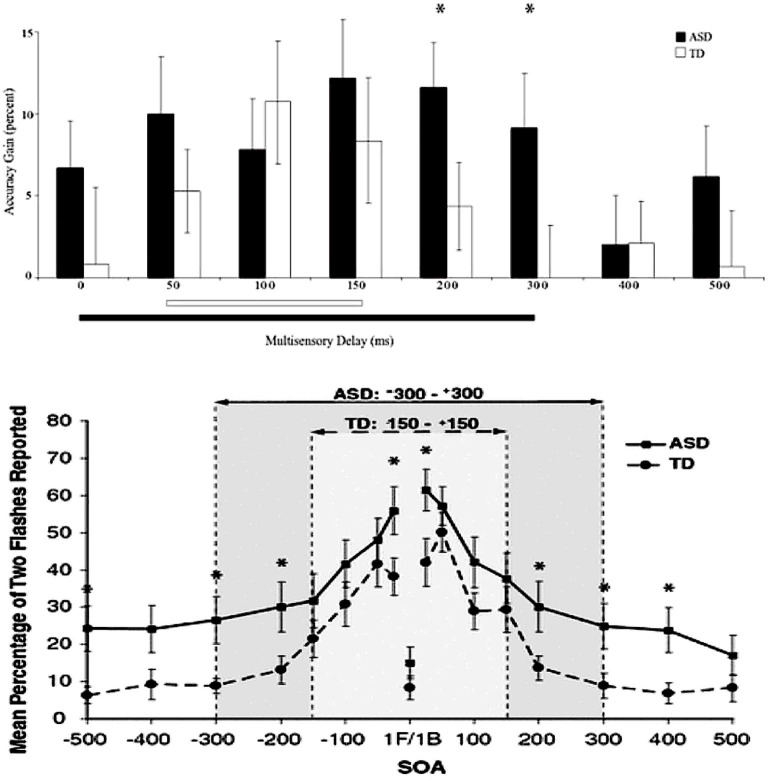
Shift in temporal binding window in multisensory integration in autism spectrum disorder (ASD). *Significant difference (*p* < 0.05).

**Figure 3:**
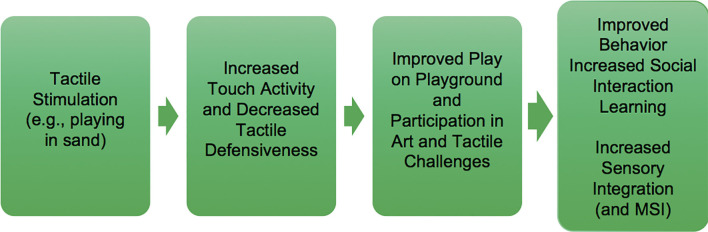
Theory of change for tactile sensory stimulation.

Stevenson et al. (2014) reported that the “window” within which the brain integrates and “binds” visual and auditory information—called auditory-visual temporal binding (approximately 100 ms in typically developing school-age children)—is highly variable and often considerably more latent (up to 500 ms or even more) in matched participants with ASD. That is, the auditory and visual sensory streams are not “integrated” within the same time frame in people with ASD. This phenomenon is depicted in Figure 2, wherein the temporal binding curve for ASD and matched control participants are overlaid on one another. This is also illustrated in Figure 3, which presents a histogram depicting the relative distribution of the temporal binding window in each group.

We hypothesize that auditory-visual temporal binding should differentially decrease for ASD under SI/SP-T because the theory of change for sensory-based treatment specifically posits that sensory integration will be improved following the delivery of these treatments. We also hypothesize that auditory-visual temporal binding will not be affected in children with ASD who are treated using applied behavioral intervention (e.g., Pivotal Response Training™; Koegel et al., 2016). A plausible theory of change including multisensory integration and use of tactile stimulation as an antecedent treatment ingredient is depicted in Figure 3.

### Controlling for Developmental Confounds

Fair and unbiased evaluation of SI/SP-T requires delivery of SI/SP-T procedures in an appropriate social and communicative developmental context (see Bialer and Miller, 2011; Miller et al., 2014), not decontextualized applications of sensory equipment, activities, and/or personal appliances such as weighted or pressure vests. While acknowledging the validity of this perspective, there exist challenges to testing the unique contributions of SI/SP-T procedures in a context that includes known *active ingredients* that are causally linked to developmental growth. For example, the aforementioned NDBI recast treatment involves language transactions that are ubiquitous in clinician-child interactions. That is, SI/SP-T conducted in naturalistic play contexts with supportive clinicians contains many known efficacious NDBI recast teaching events *in addition to* sensory events. As stated directly, social and communication elements themselves without enhanced tactile, proprioceptive, or vestibular enhancements are well established (and powerful) active ingredients in a plethora of naturalistic behavioral interventions (see Koegel et al., 1987; Cleave et al., 2015; Sandbank et al., 2020) that do not include SI/SP activities. Thus, it will be important to test whether unique treatment effects are arising from SI/SP activities and/or whether there are synergistic “value-added” contributions for SI/SP activities when implemented within the context of naturalistic social and communication intervention such as NDBIs.

As a specific example, it is well-established in the treatment literature that transactional communication exchanges facilitate language and social skills development (see National Academies of Sciences, Engineering and Medicine, 2016). The theory of change for recast treatment is based upon a naturalistic ABA approach to transactional developmental modeling (see Camarata and Yoder, 2002). Key elements for the theory of change in this naturalistic ABA approach include reinforcing attempts using social attention and natural reinforcers and pairing teaching models within meaningful communication interactions.

**Figure 4:**
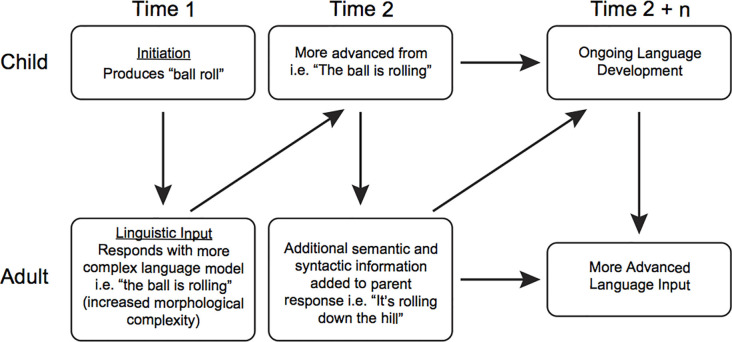
Example of language transaction.

Recast treatment and other transactional approaches (e.g., pivotal response treatment, Koegel and Koegel, 2019) incorporate transactional elements such as reinforcing and pairing in treatment sessions (see Figure 4). Stahmer et al. (2010) describe pivotal response training or pivotal response treatment as a form of naturalistic behavioral intervention based on the principles of ABA, an approach soundly supported by the scientific literature (National Research Council, 2001). Thus, transactional intervention fits within the broad rubric of evidence-based naturalistic ABA interventions that include the design, use, and evaluation of environmental modifications and interventions to produce socially significant improvement in human behavior. ABA uses antecedent stimuli (events that happen before a behavior occurs, such as a teacher asking a child what color a crayon is) and consequences (events that happen after a behavior occurs, such as giving the child the crayon after he or she names the color), to produce changes in behavior. Table 2 (from Stahmer et al., 2010) describes the key elements in the intervention.

Because of this, there is a potential confound within SI/SP-T that must be considered when conducting treatment trials; namely, fair implementation of SI/SP-T includes numerous communication transactions that are known drivers of development in typical children and in diverse populations of children with disabilities, so the unique impact of SI/SP procedures should be tested. The question is whether treatment gains associated with SI/SP-T are *differentially* associated with the *sensory* ingredients or, more broadly, to the *transactional* ingredients.

Therefore, it is important to discriminate the effects of sensory ingredients from those of transactional ingredients. A potential solution could be to deliver SI/SP-T while omitting transactions, but experts in SI/SP-T concur that this type of socially unusual intervention—wherein the clinician does not interact with a child in a normal fashion—may unfairly bias the results against SI/SP-T. Another solution is to conduct an RCT wherein one arm includes delivery of transactional treatment *with* sensory events, as compared to transactional intervention *without* sensory ingredients. This alternative approach is both practical and feasible and can be conducted with high fidelity of implementation and to test for synergistic “value-added” effects from SI/SP-T.

As a case, for example, which we acknowledge is a weak form of evidence, but none the less a useful illustration of this point, consider the following patient. A male, age 6; 3, with ASD displayed salient facial rubbing. Within the SI/SP-T theoretical framework, an OT diagnosed “sensory seeking” type sensory processing disorder and prescribed treatment using contingent sensory brushing wherein brushing on the forearm was delivered in response to facial rubbing events. Note that facial rubbing and delivery of sensory brushing are both highly salient events that were coded from video records with 100% concordance between independent coders. In addition to the sensory brushing, the clinician incidentally delivered communication transactions while sensory brushing (i.e., she interacted verbally with the child while brushing him). A counterfactual condition, wherein *transactions were delivered in the absence of brushing*, was developed and subjected to video coding for the fidelity of treatment. Naturally, coders concurred that there were no sensory events in this condition with 100% accuracy, and the concordance for communication transaction delivery was 92% (which is within the usual range of fidelity for transactional treatment, see Davis et al., 2016 as an example).

**Figure 5:**
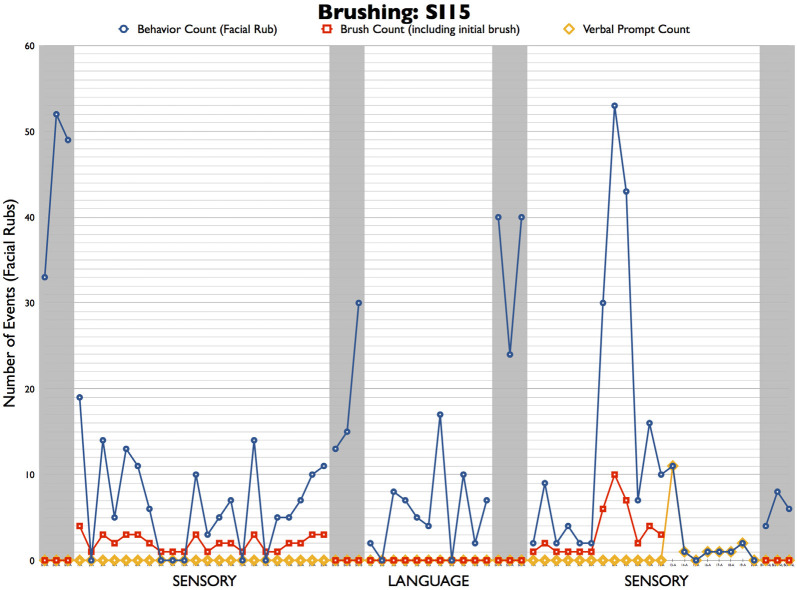
Case example illustrating confounds in sensory and transactional treatment elements.

Two different treatments–sensory brushing plus incidental communication transaction and communication transaction WITHOUT brushing–were delivered to this case using an alternating treatment design within the rubric of a single-case design (see Kennedy, 2005). Sensory brushing plus transaction was delivered first, followed by a return to baseline (no treatment) phase, then a transactional only phase, then another return to baseline (no treatment) phase, and finally, another sensory brushing phase. The results are depicted in Figure 5. The blue dots and lines represent the session counts for the “sensory seeking” facial rub events and the red squares depict the number of sensory brushing events in the session. Both conditions included an average of two communication transactions per minute. As seen in the figure, the high baseline count for facial rubbing before initiating treatment decreased during sensory brushing treatment conditions. After each treatment condition was completed, facial rub counts quickly increased during the return to baseline phases.

It is perhaps useful to examine the first baseline and treatment phases, which included sensory brushing. As can be seen, no brushing was delivered during baseline, during which time the participant exhibited a very high level of facial rubbing, ranging from 33 to 52 events per 1-h session. In the first treatment phase, the behavior decreased dramatically, falling to fewer than 20 face rubs in every session and to zero in six of the 22 sessions. A clinician keeping these data could certainly conclude that the sensory brushing was highly effective! The return to baseline phase provides further confirmation of treatment efficacy because the facial rub count immediately increased above the levels observed in treatment. However, it is important to bear in mind that sensory brushing was not the only “ingredient” delivered during this phase; incidentally, an average of two transactional events per minute during the session was provided as well when the clinician verbally interacted with the child while brushing him.

Note that in the second treatment phase, the same clinician delivered NO sensory brushing (see the red squares in phase 2) while continuing to deliver communication transactions at the same rate. As can be seen by the blue circles and line, the number of face rub events mirrored the frequency of behaviors observed in phase 1; these events decreased precipitously to below 20 per session, and on two occasions, between zero and ten events were recorded (the numbers were a little confusing without nouns) there were two at zero and six that were less than ten (but higher than zero). Again, a return to baseline yielded an increase to nearly baseline frequency of behaviors, and reinstatement of the sensory brushing treatment replicated the results from phase 1, except for a spike in face rub events during sessions 7–9. One could argue that these results suggest that communication transactions were driving the decrease in facial rub events rather than the sensory brushing. This case graphically illustrates the need to control for confounds when testing SI/SP-T.

### Summary, Conclusions, and Future Directions

SI/SP-T is a widely-used approach for treating individuals with diverse conditions and symptomology. A currently limited but emerging evidence base necessitates fair, unbiased clinical studies comparing SI/SP-T procedures to those of other established treatment approaches. This review included a presentation of one such validated NDBI treatment: Recast Treatment, which is based on a broader transactional intervention framework. Also, *multisensory integration*, broadly, and auditory-visual integration specifically, were discussed as promising approaches to *differentially* test the SI/SP-T theory of change. The article also includes a case presentation wherein confounding factors could potentially account for treatment effects that may be inaccurately attributable to an SI/SP procedure, sensory brushing, which more plausibly could be attributed to conversation transactions.

SI/SP-T is testable within the context of rigorous treatment studies, and key ingredients can be measured. Importantly, these trials should be conducted fairly and without bias to empirically evaluate the efficacy of SI/SP-T. Moreover, there has been an ongoing need for fair clinical trials of SI/SI-T. The review herein indicates that such trials can be conducted using the highest quality standards of implementation and employing objective quantitative proximal and distal measures in addition to more qualitative indices such as goal attainment scaling. Finally, these studies must be conducted using procedures that are not only faithful to the authentic implementation of SI/SP-T but also control for confounding factors. These studies should be conducted with all populations posited to benefit from SI/SP-T such as ASD, ADHD, Language Disorders, and Down Syndrome. Calls for fair studies have been appearing in the literature for more than two decades; these must be conducted soon.

## Author Contributions

SC and MTW have collaborated on the multi-sensory processing research described in this article. LM and SC have collaborated on behavioral event coding for evaluation of sensory based treatments described herein and on developing a measurable theory of change for testing sensory based intervention approaches. All authors contributed to the article and approved the submitted version.

## Conflict of Interest

The authors declare that the research was conducted in the absence of any commercial or financial relationships that could be construed as a potential conflict of interest.
